# A Case of Myelin Oligodendrocyte Glycoprotein Antibody Disease in a Pediatric Patient: Clinical Presentation, Treatment Response, and Follow-Up Considerations

**DOI:** 10.7759/cureus.70518

**Published:** 2024-09-30

**Authors:** Ahmed Elashmawy, Anas Haq, Saima Sharif, Kevin Dazy

**Affiliations:** 1 Pediatrics, Wayne State University School of Medicine, Detroit, USA; 2 Pediatrics, Children's Hospital of Michigan, Detroit, USA; 3 Neonatology, Central Michigan University College of Medicine, Detroit, USA

**Keywords:** cns demyelination, corticosteroids therapy, long-term follow up, mog antibody-associated disease, myelin-oligodendrocyte glycoprotein (mog)

## Abstract

Myelin oligodendrocyte glycoprotein antibody disease (MOGAD) is a rare autoimmune demyelinating disorder that targets the central nervous system and is characterized by antibodies that act against myelin oligodendrocyte glycoprotein (MOG). This disorder typically manifests with symptoms such as optic neuritis, transverse myelitis, or encephalitis, leading to symptoms such as vision loss, muscle weakness, sensory disturbances, and cognitive impairment. We present the case of a 10-year-old male who was eventually diagnosed with MOGAD after consideration of several other neurological and musculoskeletal disorders, who was admitted to the hospital due to an initial presentation of urinary retention and pelvic pain. The patient’s clinical course included an MRI scan showing extensive demyelinating lesions in the spinal cord, along with positive MOG antibody serology. Management involved a multidisciplinary approach, including consultations from neurology, physical therapy, and the general pediatrics team, as well as administration of high-dose corticosteroids. This case underscores the importance of early recognition and aggressive treatment of MOGAD, a disorder that can present similarly to other demyelinating diseases such as multiple sclerosis and neuromyelitis optica spectrum disorder, with the distinguishing feature of MOGAD being the presence of antibodies against MOG.

## Introduction

Myelin oligodendrocyte glycoprotein antibody disease (MOGAD) is a rare, antibody-mediated inflammatory demyelinating disorder of the central nervous system (CNS) that presents with a variety of clinical phenotypes, including optic neuritis, transverse myelitis, acute disseminated encephalomyelitis, and cortical encephalitis [1]. Although the clinical manifestations of MOGAD can resemble those of neuromyelitis optica spectrum disorder (NMOSD) or multiple sclerosis (MS), most experts consider MOGAD to be distinct with a different underlying immune pathology [1]. Myelin oligodendrocyte glycoprotein (MOG) is a myelin protein solely expressed at the outermost surface of myelin sheaths and oligodendrocyte membranes. This makes MOG a potential target of cellular and humoral immune responses in inflammatory demyelinating diseases [2].

The pathogenesis of MOGAD is centered around the discovery of a disease-specific serum immunoglobulin G antibody that selectively binds to MOG, a minor component of the myelin sheath in the CNS [3]. MOG is highly immunogenic, and although its precise function is not fully understood, it is believed to act as a cell adhesion molecule, regulate microtubule stability, and modulate immune interactions with myelin [4]. In experimental models, MOG antibodies have been shown to induce demyelination, leading to complement deposition and subsequent CNS damage [5]. Human pathology studies of MOGAD have revealed distinctive features, such as perivenous and confluent demyelination, with a notable presence of cortical and intracortical lesions [6]. The inflammatory response in MOGAD is characterized by a predominance of CD4-positive T cells and granulocytic inflammation, contrasting with the CD8-positive infiltrate typically seen in MS [6,7]. Additionally, complement deposition is a common finding, suggesting a pathologic overlap with pattern II MS, though MOGAD remains distinct, with less astrocyte damage and preserved aquaporin-4 expression compared to NMOSD [3].

The rarity of MOGAD, coupled with its overlapping symptoms with other demyelinating conditions, poses significant diagnostic challenges. Early identification and appropriate management are crucial for improving patient outcomes. Diagnosis is typically confirmed through the detection of MOG antibodies in the serum, combined with neuroimaging findings such as MRI, which often reveal demyelinating lesions in the optic nerves, spinal cord, or brain [8]. Treatment strategies for MOGAD vary depending on the severity of the initial episode and whether the disease follows a relapsing course. High-dose corticosteroids are the first line of treatment, and in cases of relapsing MOGAD, long-term immunotherapy, such as rituximab, may be required to prevent further attacks [9].

## Case presentation

A previously healthy 10-year-old male presented to the emergency department with urinary retention and pelvic pain, requiring further evaluation and management. The patient awoke early that morning with urinary urgency and groin pain. Unable to stand due to leg weakness, he crawled to the bathroom but could not urinate despite severe straining. Throughout the day, he experienced persistent leg weakness and pain with multiple unsuccessful attempts to urinate. Early in the afternoon, he managed to void a small amount of urine without blood or discoloration. His activity level and oral intake decreased significantly, with continued groin pain and multiple urination attempts. The family decided to seek emergency care due to ongoing symptoms and pain localized to the internal groin area, not the external genitalia. During physical examination, he reported abdominal pain upon palpation but denied dizziness, headache, eye pain, ear pain, foreign body insertion, loss of sensation, sharp pain, inappropriate contact with the penis, and incomplete bladder emptying prior to symptom onset. Additionally, he denied back pain, radiating leg pain, or sensory loss.

Two days before admission, the patient experienced watery red eyes, ocular pain, and fever. He was treated with erythromycin, ibuprofen, and cetirizine, resulting in improvement of his symptoms. At this time, he had minimal food intake and significant lethargy. His last normal urination and bowel movement were two days prior to the emergency department admission, with no blood, color changes, or odor alterations. He denied diarrhea or vomiting since symptom onset. Three to four weeks ago, he had a self-resolving diarrhea episode, and two weeks ago, he developed severe sinusitis, requiring a five-day course of prescribed medication. During this period, he vomited four times, but the symptoms resolved within a week.

**Figure 1:**
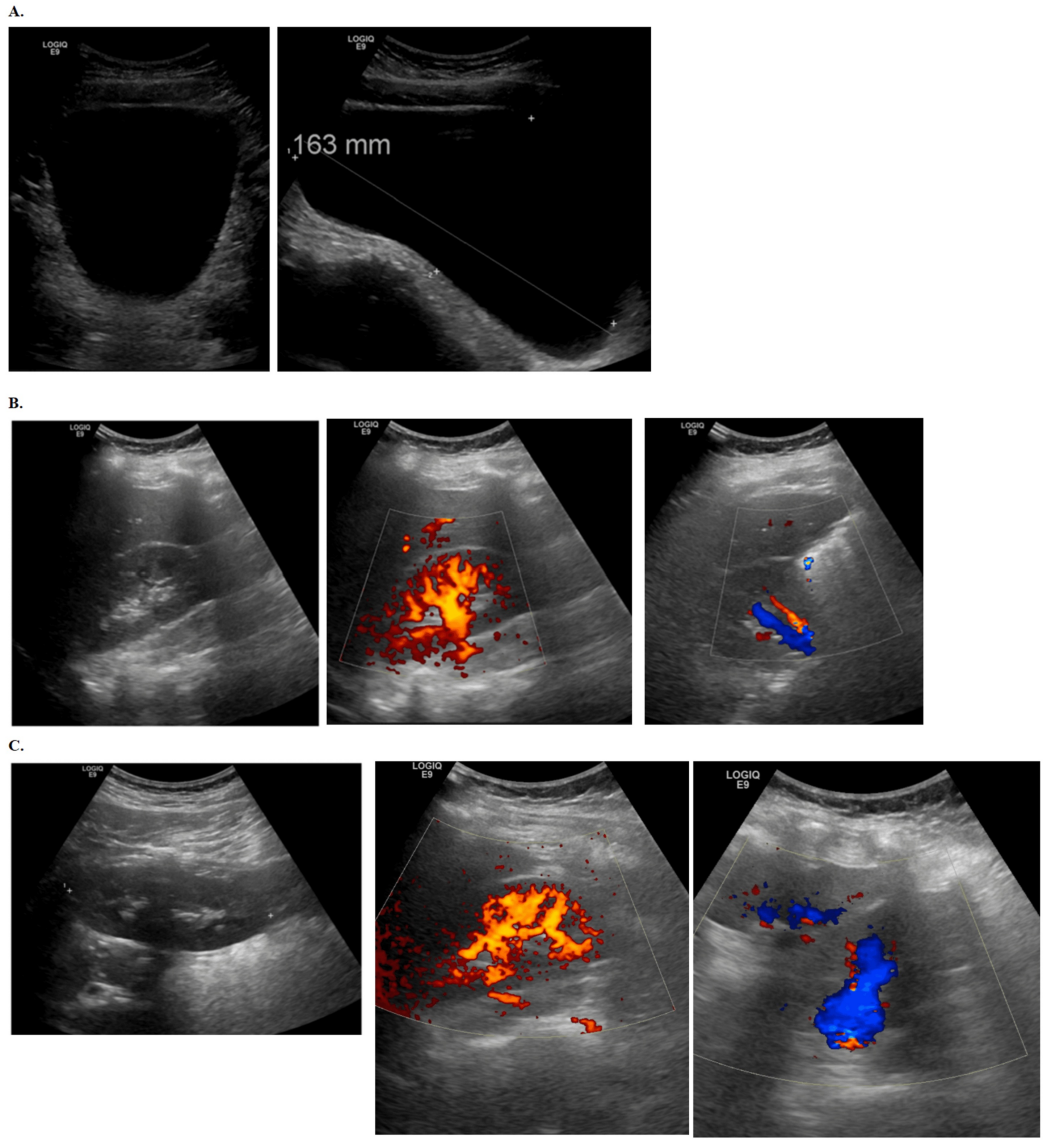
Ultrasound imaging of the bladder and bilateral kidneys A. Sagittal ultrasound view of the bladder showing the bladder wall and bladder lumen. The bladder appears slightly distended due to urine retention, with a measured vertical dimension of 163 mm. B. Transverse ultrasound views of the left kidney with color Doppler, demonstrating blood flow within the renal vessels. The images highlight the vascularity within the kidney parenchyma. C. Transverse ultrasound views of the right kidney with color Doppler. These images show the renal vasculature and confirm normal blood flow, without evidence of obstruction or vascular abnormalities. Note: The kidneys are normal in size, measuring within the range of 9-12 cm bilaterally, with smooth contours and normal parenchymal echogenicity. Corticomedullary differentiation is well maintained, and the renal collecting systems show no dilation or evidence of hydronephrosis. No renal calculi, masses, cysts, or perinephric fluid collections are seen. Color Doppler demonstrates normal renal vascular flow, with no signs of stenosis or thrombosis. No abnormalities indicative of renal parenchymal disease are present.

In the Emergency Department, he presented with a temperature of 36.9°C, blood pressure of 130/68 mmHg, heart rate of 88 beats per minute (bpm), respiratory rate of 20 breaths per minute, and an oxygen saturation of 98% on room air. Laboratory results showed a white blood cell (WBC) count of 11.5 x 10^9^/L (reference range: 4.0-11.0 x 10^9^/L), an elevated C-reactive protein (CRP) of 16.2 mg/L (reference range: <5 mg/L), a low lipase of 5 U/L (reference range: 13-60 U/L), and a slightly elevated creatinine of 0.64 mg/dL (reference range: 0.3-0.7 mg/dL for children). Urine point-of-care testing was negative and within normal limits. Abdominal and renal ultrasound imaging revealed normal kidneys without hydronephrosis or nephrolithiasis and a urinary bladder volume of 840 mL with some dilation. An abdominal ultrasound was performed (Figure 1) to assess the bladder and kidneys. The sagittal view of the bladder (Figure 1A) revealed slight distension with a vertical measurement of 163 mm, and the bladder wall appeared intact. Color Doppler imaging of the kidneys (Figures 1B, 1C) showed normal vascularity with no evidence of obstruction or abnormal flow patterns (Figure 1). Interventions included the placement of a Foley catheter, with a post-void volume unobtainable, and the administration of Tylenol for a fever of 38.0°C. The patient was then transferred to the floor for further management.

**Figure 2:**
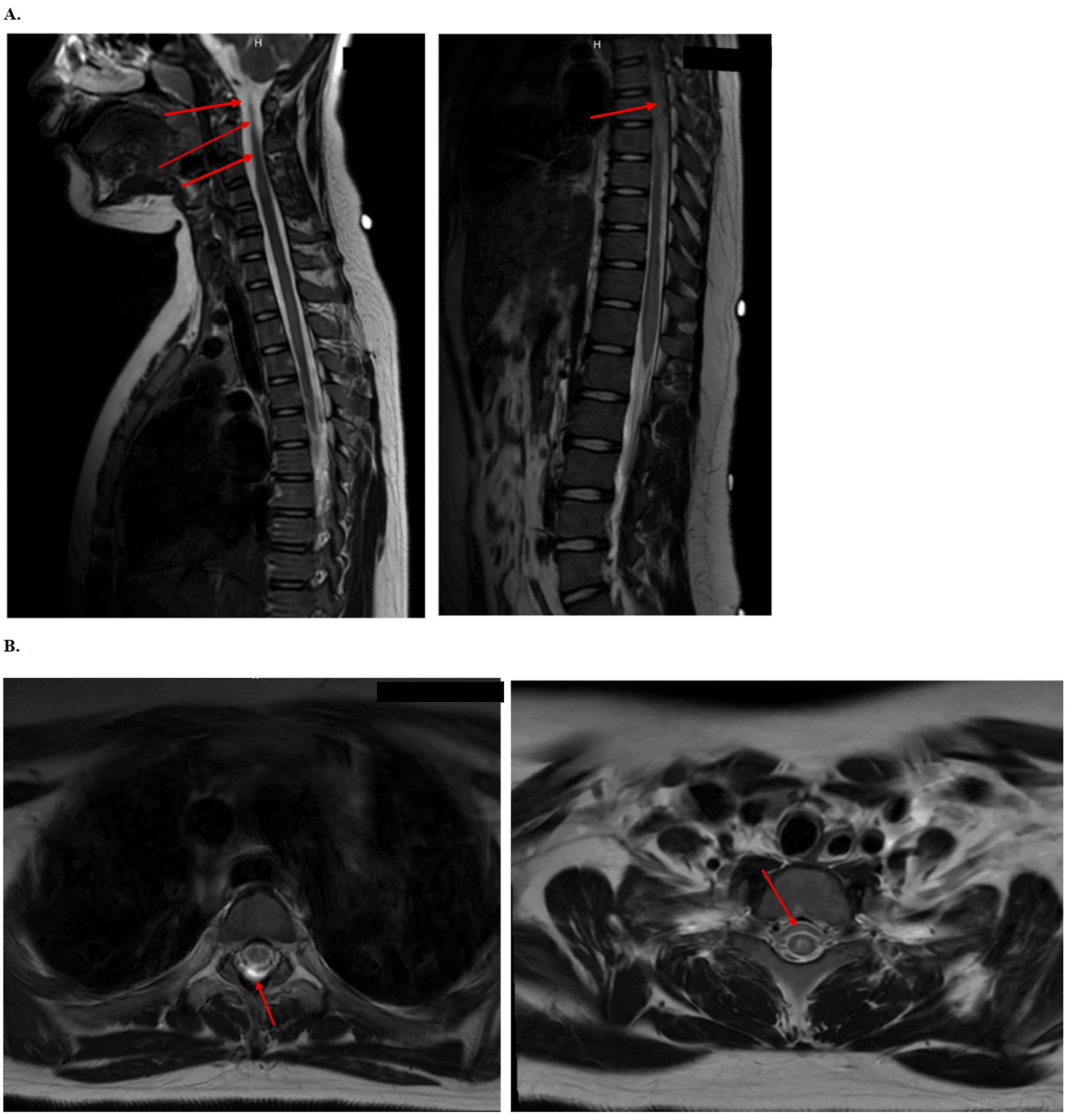
Sagittal and axial T2-weighted MRI images of the cervical, thoracic, and lumbar spine demonstrating multifocal lesions A. Sagittal T2-weighted MRI images of the cervical (left) and thoracic/lumbar (right) spine. The cervical spine image (left) demonstrates multifocal, intermittent, patchy areas of increased T2 signal intensity extending from the cervical spinal cord at C2 (uppermost arrow), as well as additional areas of involvement at C5-C6 and the thoracic spine. The thoracic/lumbar spine image (right) highlights the extent of T2 hyperintensity extending down to the conus medullaris, terminating at the superior endplate of L1. B. Axial T2-weighted MRI images at the level of C2 (left) and the thoracic spine (right). These axial images provide a detailed view of the topography and distribution of the lesions within the spinal cord, particularly at the C2 level (left image, arrow), where the lesion is characterized by increased signal intensity that is better visualized in this plane. The axial thoracic image (right) shows the corresponding transverse extent of the thoracic lesion.

Upon admission, a comprehensive workup for infectious, nutritional, and neurological causes of urinary retention, eye pain, and lower extremity weakness was initiated. Neurology and physical therapy (PT) were consulted. The nutritional workup revealed that vitamin B12 and methylmalonic acid levels were within normal limits, and serum levels of vitamins E, B6, and B1 were also normal. The neurological workup included an MRI of the cervical, thoracic, and lumbar spine, which showed multifocal, intermittent, patchy areas of increased T2 signal changes in the cervical and thoracic spinal cord between C2 and the conus medullaris, which terminates at the superior endplate of L1 (Figure 2). The craniocervical junction was normal, with no evidence of Chiari malformation. The cervical, thoracic, and lumbosacral spine alignment was well maintained, with vertebral heights, intensities, and intervertebral disc spaces appearing normal. There was no spinal canal or neural foraminal stenosis, and no disc herniation was noted. A tiny C5-C6 disc bulge was present without effacement of the anterior subarachnoid space or spinal canal stenosis. The most pronounced involvement was observed in the distal thoracic spinal cord extending into the conus medullaris, where mild expansion was noted. Cord signal abnormalities involved less than 50% of the spinal cord circumference, predominantly in the center but also affecting the lateral aspects of the cord. After gadolinium administration, subtle punctate areas of enhancement were identified in the proximal cervical spinal cord at the C3-C4 level and in the distal thoracic spinal cord extending into the conus medullaris, with no enhancement seen along the cauda equina fibers. No suspicious marrow signal changes were observed, and the conus medullaris terminated at the superior endplate of L1 without evidence of a tethered cord.

**Figure 3:**
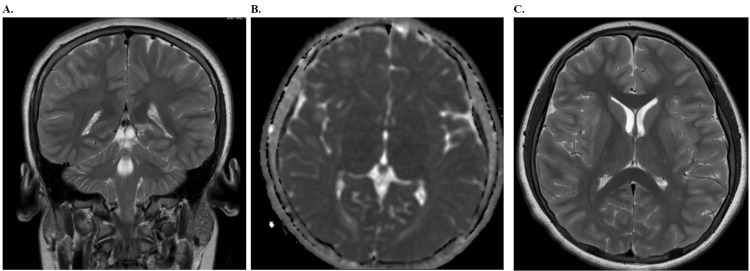
MRI characterization of white matter involvement in myelin oligodendrocyte glycoprotein antibody disease (MOGAD) in a pediatric patient A. Coronal T2-weighted image demonstrates extensive hyperintensity in the deep white matter, indicative of demyelination B. Axial diffusion-weighted imaging (DWI) shows areas of restricted diffusion, suggestive of acute inflammation C. Axial T2-weighted image reveals symmetric hyperintense lesions in the periventricular white matter, consistent with active demyelination

**Figure 4:**
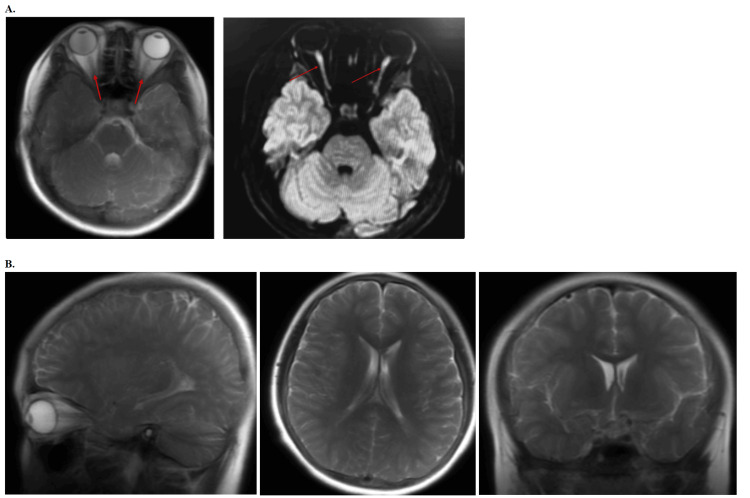
Gadolinium-enhanced T2-weighted MRI images demonstrating optic nerve and brain anatomy in a patient with a suspected neurological disorder A. Left panel shows the axial T2-weighted MRI using an FSE sequence, with gadolinium-based contrast agent enhancement, highlighting the optic nerves (indicated by red arrows). This sequence is particularly useful for detecting changes in the optic nerves and surrounding structures post-contrast administration. Right panel shows the axial T2-weighted MRI using a FLAIR sequence, post-gadolinium-based contrast, which enhances the visibility of lesions in the white matter by suppressing the cerebrospinal fluid signal. The red arrows indicate subtle areas of interest around the optic nerves. B. Left panel shows the sagittal T2-weighted MRI using an FSE sequence with gadolinium-based contrast agent enhancement, providing a detailed view of the brain’s midline structures. The T2-weighted images offer clear visualization of brain parenchyma and cerebrospinal fluid spaces, useful for identifying areas of edema or demyelination. Middle panel shows the axial T2-weighted MRI using an FSE sequence with gadolinium-based contrast agent, offering a clear depiction of the brain’s deep structures, including the ventricles and surrounding tissues. Right panel shows the coronal T2-weighted MRI using an FSE sequence with gadolinium-based contrast agent, showing a cross-sectional view of the brain and optic nerves. These T2-weighted images were obtained using a fat-suppressed sequence to enhance the visualization of the optic nerves and associated structures. FSE, fast spin echo; FLAIR, fluid-attenuated inversion recovery

The MRI of the brain further revealed multifocal, scattered T2 hyperintense lesions (Figure 3) in the supratentorial deep and subcortical white matter, including the bilateral external capsules, right optic radiation, and periventricular white matter. These findings, along with abnormal parenchymal enhancement after gadolinium administration (Figure 3), were indicative of an active demyelinating disease, with anti-MOG syndromes considered in the differential diagnosis. Limited evaluation of the optic nerves demonstrated subtle enhancement, particularly in the posterior aspect adjacent to the optic chiasm, raising concerns for underlying optic neuritis (Figure 4). A fluoroscopic-guided lumbar puncture was performed, revealing an elevated opening cerebrospinal fluid (CSF) pressure of 50 cm H_2_O (range: 6-25 cm H_2_O) and a closing pressure of 39 cm H_2_O (range: 6-25 cm H_2_O), with no bacterial growth on culture and negative fluid analysis. Approximately 18 mL of clear CSF was removed, and the patient tolerated the procedure well with no immediate postprocedural complications. The patient's diagnosis was later confirmed to be MOGAD, based on his presenting symptoms, MRI findings showing multifocal demyelinating lesions, and positive MOG antibodies identified in the cerebrospinal fluid from the lumbar puncture.

## Discussion

This report describes the case of a 10-year-old male who presented to the emergency department with urinary retention, pelvic pain, and lower extremity weakness, symptoms that were initially concerning for a range of neurological and urological conditions. The patient’s history included recent episodes of eye pain and fever, which had been treated with antibiotics and anti-inflammatory medications, but his symptoms evolved to include significant leg weakness and difficulty urinating, prompting further evaluation. MRI revealed multifocal, patchy areas of demyelination in the spinal cord, particularly in the cervical and thoracic regions, as well as subtle brain abnormalities. A positive MOG antibody serology confirmed the diagnosis of MOGAD, an autoimmune demyelinating disorder of the CNS [1]. The patient responded well to a regimen of high-dose intravenous corticosteroids, specifically methylprednisolone at 30 mg/kg/day, administered over five days. This treatment led to a significant improvement in symptoms, with only mildly abnormal deep tendon reflexes remaining at follow-up.

The clinical presentation of this patient aligns with the known spectrum of symptoms associated with MOGAD, which includes optic neuritis, transverse myelitis, and encephalomyelitis [1]. In this case, the predominant symptoms of urinary retention and lower extremity weakness were directly related to transverse myelitis [10-11], a common manifestation of MOGAD. The MRI findings of multifocal T2 hyperintense lesions, particularly in the cervical and thoracic spinal cord, are consistent with the neuroimaging characteristics typically observed in MOGAD cases [12-13]. Studies have shown that these lesions often extend longitudinally, involving multiple segments of the spinal cord, which was also observed in this patient. The involvement of the conus medullaris, with mild expansion noted on MRI, further supports the diagnosis [2]. MOGAD is often differentiated from other demyelinating diseases such as MS and NMOSD based on clinical presentation, neuroimaging findings, and serological testing. While MS typically presents with a relapsing-remitting course and NMOSD is characterized by severe attacks affecting the optic nerves and spinal cord, MOGAD can present with overlapping features but tends to have a distinct monophasic or relapsing course [14]. The detection of MOG antibodies in the serum was crucial in establishing the diagnosis in this case, as MOGAD is defined by the presence of these antibodies, distinguishing it from MS and NMOSD [2]. The importance of accurate and timely diagnosis cannot be overstated, as the management and prognosis of MOGAD differ significantly from other demyelinating disorders.

MOGAD is often differentiated from other demyelinating diseases such as MS and NMOSD based on clinical presentation, neuroimaging findings, and serological testing. In MS, brain lesions often present as well-defined, ovoid, and periventricular, with a predilection for the corpus callosum and juxtacortical regions [15]. Lesions in MS are typically small and multiple, and can show gadolinium enhancement in the acute phase. NMOSD lesions, on the other hand, are more commonly found in the optic nerves and spinal cord, often extending over three or more vertebral segments (longitudinally extensive transverse myelitis), with frequent involvement of the area postrema and dorsal medulla [16].

In contrast, MOGAD lesions are more likely to involve the deep gray matter, brainstem, and cerebellum. Optic neuritis in MOGAD typically shows more extensive nerve involvement than in MS, often affecting the anterior portion of the optic nerve. On T2-weighted MRI, these lesions are hyperintense, and post-gadolinium sequences may show less distinct or patchy enhancement compared to the more discrete enhancements seen in MS. In this case, the neuroimaging findings were consistent with MOGAD, as the lesions were predominantly located in the brainstem and optic nerves, with bilateral involvement. The imaging also demonstrated gadolinium enhancement, which, although present, was not as pronounced or sharply demarcated as typically observed in MS. These imaging features, combined with the serological presence of MOG antibodies, were crucial in distinguishing this case from MS and NMOSD [2,14].

The management of MOGAD typically involves the use of high-dose corticosteroids, which are effective in reducing inflammation and controlling acute attacks [9]. In this case, the patient was treated with high-dose corticosteroids, resulting in significant clinical improvement. The use of corticosteroids is well-supported in the literature as the first-line treatment for acute MOGAD attacks. The patient’s positive response, with a marked reduction in symptoms and stabilization of neurological function, aligns with outcomes reported in other studies. However, the potential for a relapsing course remains a concern, and long-term management may require ongoing immunosuppressive therapy [9].

In relapsing cases of MOGAD, immunotherapies such as rituximab, which targets CD20-positive B cells, have shown promise in preventing further attacks [17]. The decision to initiate long-term immunotherapy is often based on the frequency and severity of relapses, as well as the patient’s overall response to initial treatment. In this case, the plan includes monitoring the patient with repeat neuroimaging in three months and follow-up in the neuroimmunology clinic. The family has been counseled on the potential need for long-term immunotherapy if relapses occur, with rituximab being a likely option due to its efficacy in preventing B-cell-mediated autoimmune attacks.

This case highlights the critical importance of early recognition and aggressive treatment of MOGAD, particularly in pediatric patients who may present with atypical symptoms. Given the overlap of clinical features with other demyelinating diseases, clinicians must maintain a high index of suspicion for MOGAD when evaluating patients with unexplained neurological symptoms, especially when MRI findings suggest demyelination. The case also underscores the value of a multidisciplinary approach, involving specialists in neurology, PT, and pediatrics, to manage the complexities of this disease. Early intervention with corticosteroids can significantly improve outcomes, but ongoing monitoring and the potential for long-term immunotherapy must be considered in managing relapsing cases.

One limitation of this case is the potential for variability in the clinical presentation of MOGAD, which may delay diagnosis and appropriate treatment. Further research is needed to better understand the long-term prognosis of pediatric patients with MOGAD and to develop more standardized management protocols. Additionally, studies exploring the efficacy of different immunotherapeutic agents in preventing relapses and reducing long-term disability in MOGAD could provide valuable insights for improving patient outcomes. The role of biomarkers and advanced imaging techniques in predicting disease course and treatment response also warrants further investigation.

The follow-up for this patient is a critical component of managing MOGAD, given the potential for a relapsing-remitting course. After the initial treatment with high-dose corticosteroids, the patient showed significant improvement, but the risk of recurrence necessitates ongoing monitoring. The plan includes repeating neuroimaging of the brain and cervical spine in three months to assess for any new or worsening lesions, which could indicate a relapse or progression of the disease. Regular follow-up appointments in the neuroimmunology clinic are essential to evaluate the patient’s clinical status and adjust treatment as necessary. During these visits, the healthcare team will monitor for any emerging symptoms and review the need for long-term immunosuppressive therapy, such as rituximab, should the patient experience a relapse [9]. The family has been thoroughly counseled on the importance of vigilant observation for any new neurological symptoms, and they have been instructed to seek immediate medical attention if such symptoms occur. This proactive approach aims to manage the disease effectively and minimize the risk of long-term neurological impairment.

In addition to the neuroimmunology follow-up, continuity of care is paramount, especially given the chronic nature of MOGAD and the potential for recurrence. Regular follow-up visits should initially be monthly, gradually shifting to every three to six months based on the stability of the patient's condition. These visits should include detailed clinical assessments to monitor for signs of relapse, particularly new neurological symptoms or imaging changes. Systemic monitoring should include regular checks of blood pressure, blood glucose, and electrolytes to manage potential steroid side effects [18]. If relapses occur or if long-term steroid use leads to significant side effects, the introduction of steroid-sparing agents, such as methotrexate or rituximab, should be considered. Educating the patient and family about the signs of relapse and the importance of medication adherence is essential for early detection and management. Therefore, vigilant follow-up is necessary to adjust treatment as needed and prevent complications.

## Conclusions

This case report emphasizes the challenges of diagnosing and managing MOGAD in a pediatric patient. Early and accurate diagnosis, supported by neuroimaging and positive identification of MOG antibodies, was crucial for guiding effective treatment. The patient's significant improvement following high-dose corticosteroid therapy highlights the importance of early, aggressive intervention in preventing long-term neurological damage. Continuous follow-up and monitoring, including periodic imaging, are necessary due to the potential for a relapsing-remitting course. This case also contributes to the growing body of literature on MOGAD, underscoring the importance of including MOGAD in the differential diagnosis of demyelinating disorders.
